# Photon-Counting Detector CT for Imaging Around Orthopedic and Spinal Metallic Implants: A Systematic Review

**DOI:** 10.7759/cureus.117010

**Published:** 2026-09-27

**Authors:** Ahmed Y Sabi, Yazan Y Daghriri, Nasser A Abu-Alzawayid, Mohammed A Najmi, Ashjan H Shaddawi, Mohammed M Alfaifi, Faisal O Almohaily, Mohammed A Alzahrani, Khalid M Albasheit, Mohammed I Alawad, Yahya A Hakami

**Affiliations:** 1 College of Medicine, Jazan University, Jazan, SAU; 2 College of Medicine, King Khalid University, Abha, SAU; 3 College of Medicine, Najran University, Najran, SAU; 4 General Surgery, King’s College Hospital London, Jeddah, SAU; 5 College of Medicine, King Faisal University, AlHofuf, SAU; 6 Diagnostic Radiology and Nuclear Medicine, Jazan Health Cluster, Jazan, SAU

**Keywords:** image quality, metal artefact reduction, metallic implants, orthopaedic implants, photon-counting detector computed tomography, radiation dose, spectral computed tomography, spinal implants, virtual monoenergetic imaging

## Abstract

Metallic orthopedic and spinal implants degrade computed tomography (CT) image quality through beam hardening, photon starvation, and scatter artifacts that can obscure adjacent bone and soft tissue. Photon-counting detector CT (PCD-CT) provides intrinsic spectral data, electronic noise suppression, ultra-high spatial resolution, and high geometric dose efficiency, features that are theoretically well suited to imaging around metal. No systematic review has compared PCD-CT with energy-integrating detector CT (EID-CT) in this setting. PubMed/MEDLINE, Cochrane Central Register of Controlled Trials (CENTRAL), Web of Science Core Collection, and Scopus were searched on August 2, 2026, without language, date, or study-design restrictions. Eligible studies directly compared PCD-CT with EID-CT in the presence of metallic orthopedic or spinal implants in clinical, cadaveric, ex vivo, or phantom settings and reported outcomes related to image quality, metal artifacts, bone or implant-interface visualization, diagnostic confidence, image noise, or radiation dose. PCD-CT consistently reduced image noise and increased signal-to-noise and contrast-to-noise ratios in three of three studies and reduced radiation dose by approximately 45%-50% at comparable image quality in two of two direct comparisons. Bone and bone-implant interface visualization generally favored PCD-CT in three of four studies. Metal-artifact reduction was conditional: PCD-CT outperformed EID-CT when high-kiloelectron-volt (keV) virtual monoenergetic imaging (VMI), with or without iterative metal-artifact reduction (iMAR) and tin filtration, was applied, whereas PCD-CT polyenergetic images were not consistently superior and occasionally demonstrated greater streak artifacts than EID-CT. When combined with spectral post-processing, PCD-CT matches or exceeds EID-CT for imaging adjacent to orthopedic and spinal metal while approximately halving radiation dose. However, the evidence base is small and heterogeneous, and larger paired clinical studies using standardized, objective outcomes are needed.

## Introduction and background

Metallic implants are common on musculoskeletal and spinal computed tomography (CT), including joint arthroplasties, spinal instrumentation, and fracture-fixation hardware. CT is widely used to assess implant position, periprosthetic bone, fracture healing, loosening, and infection [1,2]. However, metal can substantially degrade image quality through beam hardening, photon starvation, and scatter, producing streak artifacts that obscure the peri-implant region of clinical interest [3,4].

Several metal-artifact reduction (MAR) strategies have been developed. Acquisition-based approaches include higher tube potential and tin prefiltration, whereas reconstruction- and post-processing-based approaches include iterative metal-artifact reduction (iMAR), high-kiloelectron-volt (keV) virtual monoenergetic imaging (VMI), and deep-learning-based MAR. Although these techniques can reduce artifacts, they may introduce secondary artifacts or reduce fine-detail visualization [1-4].

Photon-counting detector computed tomography (PCD-CT) represents a major advance in detector technology. Unlike energy-integrating detector computed tomography (EID-CT), which measures the total energy deposited by incoming photons, PCD-CT detects individual photons and categorizes them according to energy [3-6]. This provides intrinsic spectral information, enabling VMI without a separate dual-energy acquisition, while also reducing electronic noise and enabling smaller detector elements for high-resolution imaging. These characteristics may be particularly advantageous for imaging around metallic implants [6-10].

A growing but heterogeneous body of research has directly compared PCD-CT with EID-CT in the presence of metallic implants across phantom, cadaveric, ex vivo, and clinical settings [2,4,8]. However, the extent to which PCD-CT itself reduces metal artifacts, as opposed to the contribution of spectral post-processing, remains unclear. No systematic review has specifically synthesized direct comparisons of PCD-CT and EID-CT for imaging around orthopedic and spinal metallic implants. This systematic review therefore aimed to identify and synthesize studies comparing PCD-CT with EID-CT for assessment of bone and soft tissue adjacent to metallic orthopedic and spinal implants, focusing on metal-artifact severity, image quality, bone and bone-implant interface visualization, diagnostic confidence, image noise, and radiation dose.

## Review

Methodology

Reporting

This systematic review was conducted and reported in accordance with the Preferred Reporting Items for Systematic Reviews and Meta-Analyses (PRISMA) 2020 statement [11].

Eligibility criteria

Studies were eligible if they directly compared PCD-CT with EID-CT using the same subjects, specimens, or phantoms; involved metallic orthopedic or spinal implants within the CT field of view; reported original clinical, cadaveric, ex vivo, or phantom data; and reported at least one outcome related to metal-artifact severity, image quality, bone or bone-implant interface visualization, diagnostic confidence, image noise, signal-to-noise ratio (SNR), contrast-to-noise ratio (CNR), or radiation dose. We excluded reviews, studies without a direct EID-CT comparator, studies of non-orthopedic or non-spinal implants, and algorithm-development studies without an eligible image comparator. We applied no restrictions on language, publication date, or study design.

Information Sources and Search Strategy

PubMed/MEDLINE, the Cochrane Central Register of Controlled Trials (CENTRAL), Web of Science Core Collection, and Scopus were searched on August 2, 2026. The primary search combined terms for PCD-CT with terms for metallic implants and metal artifacts; comparator terms for EID-CT were omitted to maximize sensitivity and were verified during full-text screening. Reference lists of included studies and relevant reviews were also screened for additional records.

Study Selection and Data Extraction

After deduplication, two reviewers (NAA-A and AYS) independently screened titles and abstracts and assessed potentially eligible full texts. Disagreements were resolved by discussion or, when necessary, by a third reviewer (YYD). Two reviewers (NAA-A and AYS) independently extracted data using a standardized form, with discrepancies resolved by a third reviewer (YYD). Extracted data included study design and setting, sample size, implant site and material, scanner models, acquisition parameters and radiation dose, reconstruction techniques, including polyenergetic imaging, VMI, iMAR, and tin filtration, as well as reader characteristics, rating scales, and qualitative and quantitative outcomes.

Risk-of-Bias Assessment

Risk of bias was assessed using a modified Quality Assessment of Diagnostic Accuracy Studies 2 (QUADAS-2) tool adapted for paired imaging comparisons, covering participant or specimen selection, conduct and interpretation of PCD-CT and EID-CT, flow and timing, and applicability [12]. The Risk of Bias in Non-randomized Studies of Interventions (ROBINS-I) tool was additionally applied to clinical studies to assess potential confounding. Phantom, cadaveric, and ex vivo studies were assessed using the modified QUADAS-2 tool only [13].

Results

Study Selection

The searches identified 2,173 records: PubMed/MEDLINE (*n *= 301), Cochrane Central Register of Controlled Trials (*n* = 24), Web of Science (*n* = 954), and Scopus (*n* = 894). After removal of 390 duplicates, 1,783 records were screened, and 30 full-text reports were assessed. Twenty-four were excluded because they were reviews or lacked original comparative data (*n* = 15), involved off-scope implant sites (*n* = 5), or lacked an eligible PCD-CT versus EID-CT comparison (*n* = 4). Six studies met the eligibility criteria (Figure 1) [1-6].

**Figure 1:**
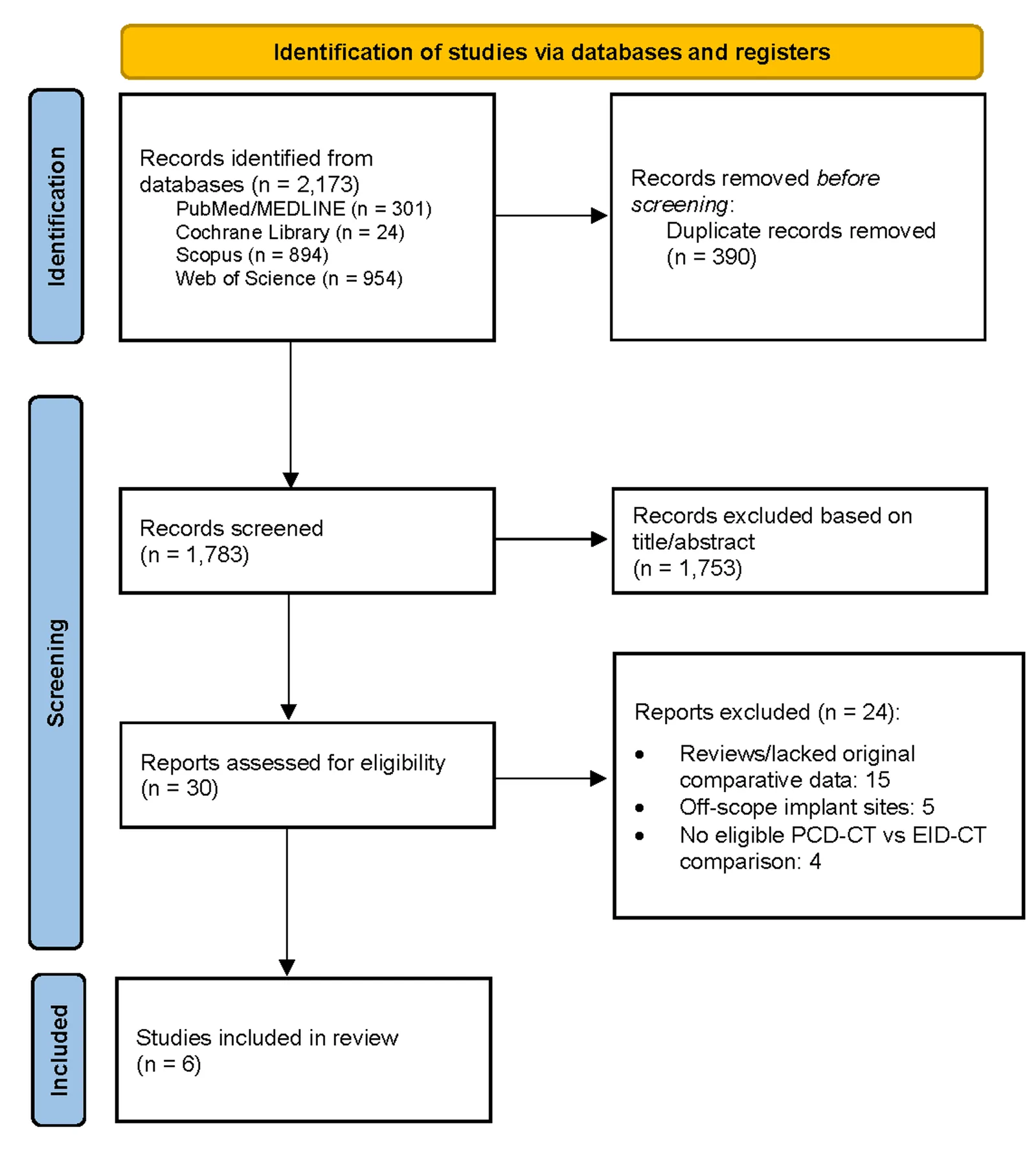
PRISMA flow diagram depicting the study selection process for the systematic review. PRISMA (Preferred Reporting Items for Systematic Reviews and Meta-Analyses) flow diagram detailing the identification, screening, eligibility assessment, and inclusion of studies in the final synthesis.

Study Characteristics

The six studies comprised one combined phantom and clinical study, one cadaveric study, one ex vivo study, and three clinical studies [1-6]. Implant sites included the spine, hip, wrist, and forearm. Five studies used a first-generation commercial PCD-CT system (NAEOTOM Alpha) [2-6], whereas one used a research prototype [1]. Comparisons included polyenergetic PCD-CT, high-keV VMI, iMAR, and tin filtration [1-6] (Table 1).

**Table 1:** Characteristics of the six included studies. Characteristics of the six studies directly comparing PCD-CT with EID-CT for imaging around metallic orthopedic and spinal implants. The table summarizes study design and setting, sample and implant characteristics, CT scanner models, acquisition parameters and radiation dose, reconstruction techniques, reader characteristics and rating scales, and assessed outcomes. CNR, contrast-to-noise ratio; CT, computed tomography; CTDIvol, volume computed tomography dose index; EID-CT, energy-integrating detector computed tomography; HU, Hounsfield units; iMAR, iterative metal-artifact reduction; PCD-CT, photon-counting detector computed tomography; SNR, signal-to-noise ratio; STD, standard; UHR, ultra-high-resolution; VMI, virtual monoenergetic imaging.

Study	Design and setting	Sample	Implant	PCD-CT scanner	EID-CT comparator	Acquisition (dose)	Reconstructions compared	Readers and scale	Outcomes assessed
Zhou et al., 2019 [1]	Phantom (3D-printed spine with pedicle screws) and prospective clinical study	20 patients	Spinal pedicle screws	Prototype dual-source photon-counting detector CT (PCD-CT; SOMATOM CounT)	Clinical energy-integrating detector CT (EID-CT; SOMATOM Flash/Sensation)	Tin filtration; approximately 12–21 mGy	PCD-CT high-energy-threshold (tin) versus standard EID-CT	3 radiologists; 5-point scale	Artefact severity; image noise; cortex, trabeculae, and implant–bone interface visualization; diagnostic confidence
Rau et al., 2023 [2]	Prospective clinical study (17 prior EID-CT examinations and 15 matched examinations)	32 patients (10 with implants)	Spinal hardware	NAEOTOM Alpha	SOMATOM Definition (matched EID-CT)	CTDIvol: 8.83 versus 15.7 mGy	PCD-CT 65- and 130-keV VMI (Br62/64) versus EID-CT	4 radiologists; 5-point Likert scale	Image quality; sharpness; artefacts; noise; diagnostic confidence; HU within artefacts; radiation dose
Woisetschläger et al., 2023 [3]	Ex vivo comparative study	2 acetabular cups (cemented and uncemented)	Acetabular cups	NAEOTOM Alpha	SOMATOM Force	Equal dose (approximately 12.2 mGy); 140 kVp	Multiple kernels (Br76–Ur98); PCD-CT versus EID-CT	4 radiologists; Likert scale	Bone–implant and bone–cement interface delineation (subjective and quantitative)
Fukuda et al., 2024 [4]	Cadaveric study with standardized parameters	4 forearms (1 with a radial plate)	Radial plate	NAEOTOM Alpha	5 EID-CT scanners (Drive, Force, Flash, Definition AS, X.cite)	Standardized: 3.83/4.99 mGy	Sn−/Sn+/120-keV VMI; STD and UHR modes	3 radiologists; 5-point scale	Bone and fracture visibility; image quality; noise; SNR; CNR; streak-artefact area
Kämmerling et al., 2025 [5]	Prospective clinical study	15 patients	Titanium wrist prostheses	NAEOTOM Alpha	SOMATOM Force	Matched clinical protocols	PCD-CT polyenergetic and 130-keV VMI versus EID-CT polyenergetic (Br76/89)	5 radiologists; 7-point scale	Bone visualization; bone–metal interface interpretability; streak artefacts
Marth et al., 2026 [6]	Retrospective clinical study (26 with both examinations)	100 examinations (50 EID-CT and 50 PCD-CT)	Lumbar instrumentation	PCD-CT (NAEOTOM)	Standard EID-CT	Thin filtration; CTDIvol: 6.4 versus 12.5 mGy	Standard EID-CT versus standard PCD-CT, VMI120, iMAR, and VMI120 + iMAR	2 readers; 4-point Likert scale	Artefact extent; cortical and trabecular visibility; silhouette and dark-zone artefacts; HU; radiation dose

Risk of Bias

No study was considered to have uniformly low risk of bias. Common limitations included small samples, subjective reader-based outcomes, and incomplete pairing of PCD-CT and EID-CT acquisitions in clinical studies. Using the modified QUADAS-2 tool, most studies had some concerns. One retrospective, predominantly unpaired clinical study had high overall risk [6], while a two-specimen study raised concerns regarding selection [3]. Using the ROBINS-I tool, two clinical studies had moderate risk, and one had serious risk, primarily because of confounding (Tables 2-3) [4-6].

**Table 2:** Risk of bias assessed using the modified QUADAS-2 tool. Risk of bias was assessed across study selection, conduct and interpretation of the PCD-CT index test, conduct and interpretation of the EID-CT comparator, and flow and timing, with additional assessment of applicability. +, low concern; ?, some concerns or unclear; ✕, high concern. Overall risk of bias reflects the highest concern across the assessed domains. QUADAS-2, Quality Assessment of Diagnostic Accuracy Studies 2; PCD-CT, photon-counting detector computed tomography; EID-CT, energy-integrating detector computed tomography

Study	D1: Selection	D2: Index test (PCD-CT)	D3: Comparator (EID-CT)	D4: Flow and timing	Applicability	Overall
Zhou et al. [1]	?	?	+	+	✕	?
Woisetschläger et al. [3]	✕	+	+	+	?	?
Fukuda et al. [4]	?	?	+	+	?	?
Kämmerling et al. [5]	?	?	+	+	+	?
Marth et al. [6]	✕	?	?	✕	+	✕

**Table 3:** Risk of bias assessed using ROBINS-I. Risk of bias in clinical studies was assessed using the Risk of Bias in Non-randomized Studies of Interventions (ROBINS-I) tool across confounding, selection of participants, classification of interventions, deviations from intended interventions, missing data, measurement of outcomes, and selection of the reported result. +, low risk of bias; ?, moderate risk of bias; ✕, serious risk of bias. Overall risk of bias reflects the worst domain rating. ROBINS-I, Risk of Bias in Non-randomized Studies of Interventions

Study	D1: Confounding	D2: Selection	D3: Classification	D4: Deviations	D5: Missing data	D6: Outcome measurement	D7: Reported result	Overall
Rau et al. [2]	?	?	+	+	+	?	+	?
Kämmerling et al. [5]	+	?	+	+	+	?	+	?
Marth et al. [6]	✕	?	+	+	?	?	+	✕

Metal-Artifact Severity

Six studies assessed metal-artifact severity. PCD-CT consistently reduced artifact relative to EID-CT when high-keV VMI was used [1-5]. High-energy-threshold or tin-filtered PCD-CT reduced streak artifact and improved reader preference, while 130-keV PCD-CT VMI reduced artifact compared with lower-keV reconstructions [1,4]. In contrast, PCD-CT polyenergetic images produced greater streak artifact than EID-CT in two studies, whereas high-keV VMI reduced artifact compared with both PCD-CT polyenergetic and EID-CT images [2,5]. Combined VMI and iMAR provided the greatest artifact reduction in one clinical study [6]. Overall, high-keV VMI favored PCD-CT in four of five studies [1,2,4,5], whereas polyenergetic PCD-CT alone was not consistently superior to EID-CT [2,5].

Bone and Bone-Implant Interface Visualization

Four studies assessed bone or bone-implant interface visualization, with results generally favoring PCD-CT [1-3,5]. PCD-CT provided better visualization of cortical bone, trabeculae, and implant-bone interfaces in several studies, with sharper interfaces and higher reader scores than EID-CT [1,3]. One cadaveric study found no significant difference after acquisition parameters were standardized [2]. Aggressive metal-artifact reduction, particularly combined high-keV VMI and iMAR, could reduce cortical and trabecular visibility, indicating a trade-off between artifact suppression and preservation of fine detail [6].

Image Noise, SNR, and CNR

Three studies evaluated image noise or signal metrics, all favoring PCD-CT [1,2,4]. PCD-CT produced lower image noise and higher SNR and CNR than EID-CT, including in standard- and ultra-high-resolution acquisitions [1,2]. One clinical study also reported greater image sharpness with PCD-CT [4].

Diagnostic Confidence

Two studies assessed diagnostic confidence, both favoring PCD-CT [1,4]. PCD-CT was associated with greater reader confidence, and 130-keV VMI provided higher confidence than lower-keV reconstructions in the implant subgroup [4]. However, the evidence for this outcome was limited.

Radiation Dose

PCD-CT achieved comparable image quality at substantially lower radiation doses in the clinical comparisons. One study reported a volume CT dose index of 8.83 mGy for PCD-CT versus 15.7 mGy for EID-CT, while another reported median values of 6.4 versus 12.5 mGy, corresponding to reductions of approximately 45%-50% [4,6]. Cadaveric and ex vivo studies used equal or standardized doses, supporting the potential dose-efficiency advantage of PCD-CT [2,3].

Limitations

The main limitations were the small number of eligible studies, small samples, heterogeneous designs and outcome measures, and predominantly subjective assessments. One study used a research-prototype PCD-CT system, limiting generalizability. The absence of standardized effect measures precluded meta-analysis, and the synthesis relied on vote-counting by direction of effect. The search also did not include Embase or gray literature, potentially resulting in missed studies.

## Conclusions

When combined with spectral post-processing, PCD-CT can provide high-quality imaging around metallic orthopedic and spinal implants while reducing image noise and radiation exposure. High-keV VMI may further improve metal-artifact suppression, although reconstruction settings should be optimized to preserve fine anatomical detail. Current evidence remains limited, highlighting the need for larger, well-designed clinical studies using objective, task-based outcomes to determine the clinical value of PCD-CT for imaging around metallic implants.
